# Synthesis and Characterization of a New Functional Photoinitiator for the Curing of Biocompatible Polymer Materials

**DOI:** 10.3390/ma19183985

**Published:** 2026-09-19

**Authors:** Xuejiao Shang, Guoyao Li, Ziliang Yang, Rong Zhong

**Affiliations:** Department of Materials and Chemistry, School of Environmental & Chemical Engineering, Nanchang Hangkong University, Nanchang, 330063, China; 2402085600317@stu.nchu.edu.cn (X.S.); 2202077600027@stu.nchu.edu.cn (G.L.); 24020856000347@stu.nchu.edu.cn (Z.Y.)

**Keywords:** Photoinitiator, acetylsalicylic acid, photopolymerization, migration, biomaterials

## Abstract

In UV curing technology, traditional photoinitiators are widely used in fields such as food packaging and medical devices, but they have some issues. These include migration, volatility and toxicity of the residue photoinitiator during their useful periods. This study aims to develop specialized photoinitiators specifically for food packaging and medical devices to address this gap. A novel photoinitiator, 2-{2-[4-(2-hydroxy-2-methylpropan-oyl) phenoxy]ethoxy}ethyl 2-acetoxybenzoate (AI-2959), was synthesized via esterification between acetylsalicylic acid (aspirin) and the commercial photoinitiator 2-hydroxy-4′-(2-hydroxyethoxy)-2- methyl- propiophenone (I-2959). The structure was confirmed using Fourier-transform infrared spectroscopy (FTIR), proton nuclear magnetic resonance (^1^H NMR), and elemental analysis, and the physicochemical properties of AI-2959 and I-2959 were compared. The ultraviolet spectra revealed absorption characteristics similar to I-2959 but with a higher molar extinction coefficient, indicating superior photosensitivity. Thermogravimetric analysis demonstrated improved thermal stability, and photopolymerization kinetics studies showed enhanced resistance to oxygen inhibition and higher double-bond conversion. Coating performance tests indicated a shorter curing time, greater hardness, and excellent adhesion. Importantly, the relative migration rate of AI-2959 was only 10.2% of that of I-2959, significantly reducing potential biotoxicity risks. These properties suggest the good potential of AI-2959 for use in biomedical and pharmaceutical applications involving UV curing.

## 1. Introduction

UV curing technology has gained widespread application across various fields, including coatings [1], electronics and optoelectronics [2,3], adhesives [4], inks [5], composites [6,7], additive manufacturing [8], optical devices [9], and biomedical engineering [10,11], due to its high efficiency, energy conservation, and environmentally friendly characteristics. As the key component of this technology, photoinitiators absorb light energy and generate active species to initiate monomer polymerization, thereby determining the curing efficiency and final properties of the material [12,13]. However, a significant limitation of conventional photoinitiators is the potential migration of small molecules after curing [14]. This issue not only compromises the long-term stability of the material but also poses cytotoxicity risks. For example, many photoinitiator residues were detected in packaged milk [15], limiting their use in medical applications requiring high biocompatibility [16,17]. Consequently, novel photoinitiators that combine high initiation efficiency with low migration characteristics have become an important research focus, with the goal of meeting the comprehensive requirements for safety, efficiency, and performance in UV-curable biomaterials [18,19,20,21,22,23]. I-2959 (Irgacure 2959) is widely used in the preparation of biomaterials due toits favorable water solubility and relatively low cytotoxicity [24,25,26]. However, its application in biomedical fields is limited by moderate photoinitiation efficiency, a high small-molecule migration rate, and potential biotoxicity [27,28]. In recent years, molecular structure modification has emerged as an effective strategy to enhance thermal stability, improve photo-efficiency, and reduce migration [29,30,31]. One promising approach involves designing macromolecular or polymerizable photoinitiators [32,33]. Acetylsalicylic acid (aspirin), a classic analgesic and anti-inflammatory pharmaceutical, offers a valuable structural motif for such modifications [33]. The carboxylic acid group within its molecular structure provides a reactive site for esterification [34]. By incorporating the aspirin moiety into a photoinitiator design, it is possible to retain essential photochemical activity while significantly increasing themolecular size and polarity of the resulting compound [35,36]. This structur al enhancement effectively hinders migration behavior within cured polymer networks [36].

**Scheme 1:**
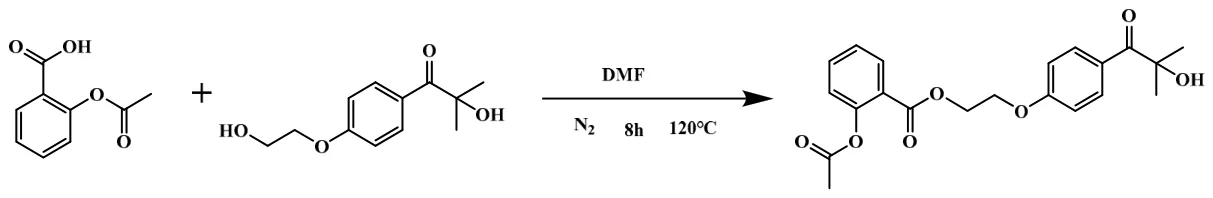
Synthetic route to AI-2959.

In this work, a novel modified photoinitiator, AI-2959, was designed and synthesized from I-2959 and acetylsalicylic acid via esterification. The goal was to merge the biocompatibility of aspirin with the photochemical characteristics of I-2959, creating a photoinitiator with a high initiation efficiency, superior thermal stability, and low migration rate. The photophysical properties, thermal stability, polymerization kinetics, and biocompatibility of AI-2959 were comprehensively evaluated. The results indicate that AI-2959 maintains high photosensitivity while significantly reducing migration, demonstrating excellent overall performance and potential in bio-safe UV-curable materials. The molecular structure and synthesis route of AI-2959 are shown in Scheme 1. A novel modified photoinitiator, 2-{2-[4-(2-hydroxy-2-methylpropionyl) phenoxy]ethoxy}ethyl 2-acetoxybenzoate (AI-2959), was synthesized through the esterification of aspirin (acetylsalicylic acid) and the commercial photoinitiator 2-hydroxy-4′-(2-hydroxyethoxy)-2-methylpropiophenone (I-2959). The experimental results indicated that the small-molecule migration rate of AI-2959 was significantly lower than that of I-2959.

## 2. Experimental

### 2.1. Instruments and Reagents

A KQ3200E ultrasonic cleaner and a VERTEX70 type Fourier-transform infrared spectrometer, equipped with a KBr test paper cassette (Bruker, Saarbrücken, SAAR, Germany), were employed in this study. A UV-1900i ultraviolet–visible spectrophotometer (Shimadzu Laboratory Equipment Co., Ltd., Kyoto, Japan) was used for spectroscopic analysis. The UV–Vis absorption spectra of I-2959 and AI-2959 were recorded in acetonitrile. Due to the different absorption intensities of the two photoinitiators, appropriate concentrations were selected to ensure reliable spectral acquisition and avoid excessive absorbance. The molar extinction coefficients were calculated according to the Beer–Lambert law to evaluate and compare the intrinsic light absorption abilities of I-2959 and AI-2959. A TG-209 Thermogravimetric Analyzer (Mettler-Toledo International Inc., Greifensee, Switzerland) and a RE-25A rotary evaporator (Shimadzu, Kyoto, Japan) were used for thermal stability experiments, and aUV-T-400W portable UV curing light (Dongguan Good Jia Machinery Equipment Co., Ltd., Dongguan, China) was used for polymerization studies.

Acetylsalicylic acid, N,N-dimethylformamide (DMF), and methane sulfonic acid were purchased from Shanghai Yingxin Laboratory Equipment Co., Ltd. (Shanghai, China). 2-Hydroxy-4-(2-hydroxyethoxy)-2-methylpropiophenone (I-2959) and dichloromethane were purchased from Shanghai Yingxin Laboratory Equipment Co., Ltd. (Shanghai, China). Purity ≥ 99%methanol was supplied by Jiangsu Xinsu New Materials Co., Ltd. (Suzhou, China). Ethyl acetate and sodium bicarbonate were purchased from Xilong Scientific Co., Ltd. (Shantou, China)**.** Anhydrous magnesium sulfate was purchased from Jiangsu Congzhong Chemical Co., Ltd. (Suzhou, China). Sodium chloride was obtained from Shanghai Aladdin Biochemical Technology Co., Ltd. (Shanghai, China); hydrochloric acid and acetonitrile were purchased from Aladdin Reagent Co., Ltd. (Shanghai, China); and industrial-grade trimethylolpropane triacrylate (TMPTA monomer) was procured from Guangzhou Bosheng New Materials Technology Co., Ltd. (Guangzhou, China). All reagents were used without purification.

### 2.2. Synthesis of AI-2959

A 250mL three-neck flask was equipped with a condenser, dropping funnel, and thermometer and kept under a nitrogen atmosphere. Acetylsalicylic acid (0.79 g, 0.01 mol) was dissolved in 60 mL of N,N-dimethylformamide (DMF) under continuous N_2_ flow. I-2959 (0.91 g, 0.02 mol) was added, followed by dropwise addition of methane sulfonic acid (0.05 g). The reaction was carried out under reflux at 110 °C for 8 h with stirring. The solvent was removed using rotary evaporation, yielding a viscous crude product. This product was dissolved in 40 mL of dichloromethane and washed sequentially with saturated brine (3 × 60 mL) and saturated NaHCO_3_ solution (3 × 60 mL). The organic phase was dried over anhydrous MgSO_4_ and purified via silica gel column chromatography, using a mixture of dichloromethane/methanol (250:1) as the eluent. A pale-yellow ester compound was obtained (0.65 g, yield: 60.2%).

### 2.3. Coating Preparation

Coatings were prepared under light-free conditions. AI-2959, 184, and I-2959 (each 5%) were dissolved in ethyl acetate and mixed with 95% TMPTA monomer. The mixture was stirred magnetically for 30 min, degassed in an ultrasonic bath (40 kHz) for 30 min, and allowed to rest for another 30 min. Using a #35 wire bar coater, uniform coatings were applied onto glass slides with a wet film thickness of 25 ± 2 μm. UV curing was performed under a nitrogen atmosphere using a handheld UV curing unit equipped with a high-pressure mercury lamp (Dalian Ruixin Co. Ltd., Dalian, China) (main wavelength of 365 nm; power density of 400 W/m^2^; lamp-to-substrate distance of 5.5 cm).

### 2.4. Coating Performance Evaluation

The coating performance was evaluated using the curing degree, pencil hardness, adhesion, surface drying time, and acid/alkali resistance as the criteria. The curing degree was evaluated using the gel content method, measuring the mass of insoluble residue after extracting the cured films in acetone. The pencil hardness test was conducted according to the ASTM D3363-22 standard [37]. A pencil was scratched five times on the film at a certain gravity (500 g or 750 g), and the hardness of the paint film was the pencil hardness that produced no scratch. The adhesion properties of the prepared coatings were evaluated using the cross-cut test according to ASTM D3359 23 standard [38]. The test results are classified into grades from 0 B (poor) to 5 B (excellent), where 5 B indicates that the edges of the cuts are completely smooth and none of the squares of the coating are detached.

### 2.5. Residual Migration Test

Coatings containing 5% AI-2959 or I-2959 in TMPTA were prepared on glass slides (wet thickness: 25 ± 2 μm) and cured in air using a handheld UV device (power density of 400 W/m^2^; distance of 5.5 cm). The cured films were cut into 1 × 1 mm fragments, and 0.08 g of each sample was dissolved in 20 mL of acetonitrile. Extraction was performed ona constant-temperature shaker (25 °C, 120 rpm) for 24 h. The extract was filtered through a 0.22 μm organic membrane, and absorbance (A) at λ_max_ was measured using a UV-1800PC spectrophotometer. The migration rate was calculated using the following equations:

(1) c=A/(ε×b)

(2) R=c(AI˗2959)/c(I˗2959)

*c* is the concentration of the photoinitiator (mol/L); *A* is the absorbance at a characteristic wavelength; *ε* is the molar extinction coefficient (L/mol·cm); *c*(AI-2959) is the concentration of AI-2959 in the extracted solution; *c*(I-2959) is the concentration of I-2959 in extracted solution; and *R* is the relative migration rate of AI-2959.

### 2.6. Photopolymerization Kinetics Measurement and Conversion Calculation

The photopolymerization kinetics of TMPTA initiated by AI-2959, I-2959, and photoinitiator 184 were investigated using real-time Fourier-transform infrared spectroscopy (RT-FTIR). The formulations were prepared by dissolving 5 wt% photoinitiator in TMPTA monomer under dark conditions. The obtained mixtures were placed between two polyethylene films and irradiated with a 365 nm UV light source under a nitrogen atmosphere. The decrease in the absorption intensity of the acrylate C=C stretching vibration peak at approximately 810 cm^−1^ was monitored during UV irradiation.

The double-bond conversion (DC) was calculated according to the following equation:

(3) $$DC\left(\%\right)=\left(1-\frac{A_{t}/A_{ref}}{A_{0}/A_{ref}}\right)\times 100$$

where $A_{0}$ and $A_{t}$ represent the integrated absorbance of the acrylate C=C bond before irradiation and after irradiation at time t, respectively. $A_{ref\;}$ represents the absorbance of an internal reference peak that remains unchanged during polymerization. The conversion rate was calculated based on the relative decrease in the C=C bond absorption intensity during UV irradiation.

## 3. Results and Discussion

### 3.1. FTIR Spectral Analysis

**Figure 1:**
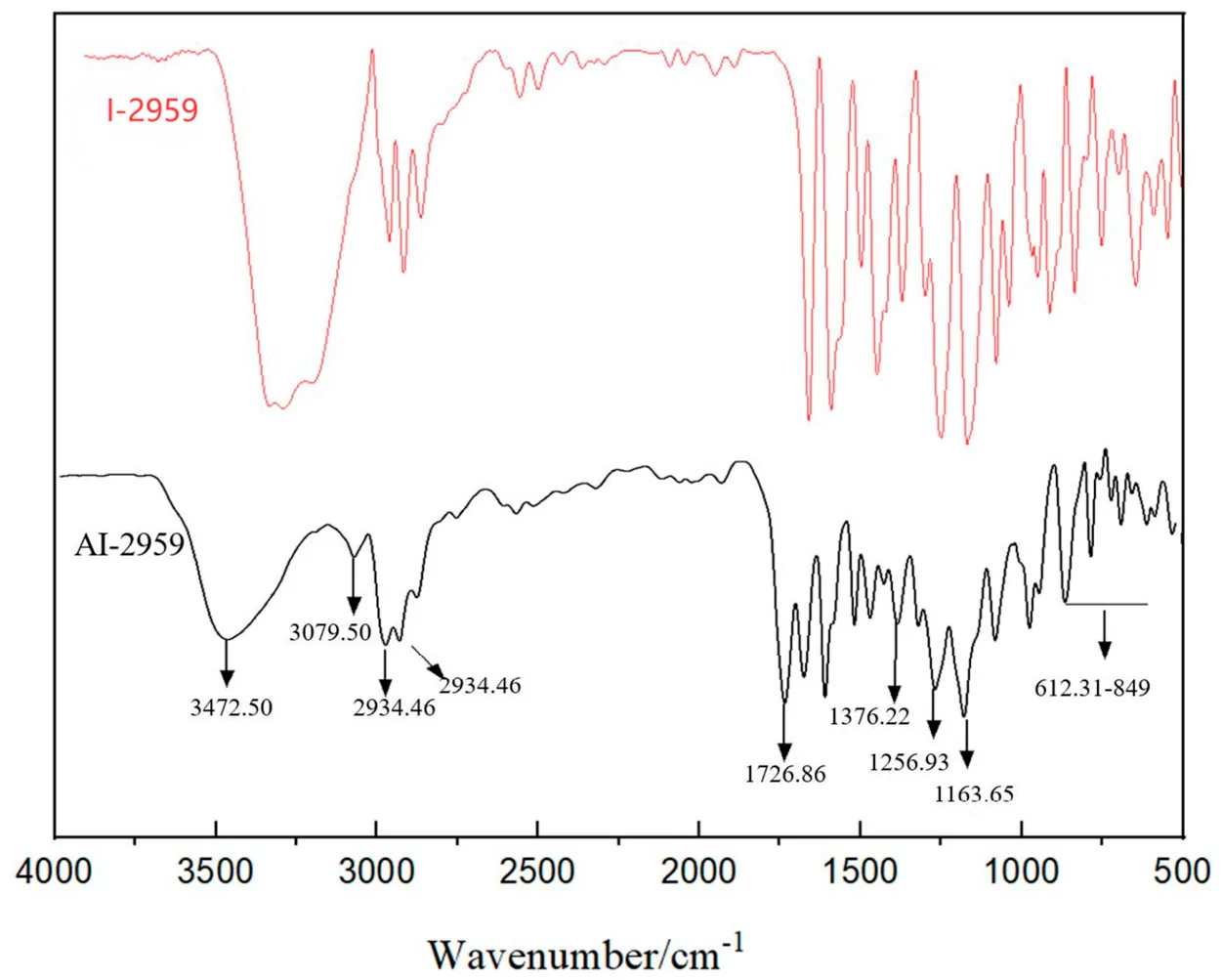
FTIR spectra of AI-2959 and I-2959.

The FTIR spectra were recorded in the range of 4000–500 cm^−1^, which covers the characteristic vibration regions of the functional groups involved in the synthesis of AI-2959. The selected range is sufficient for identifying the hydroxyl, carbonyl, aromatic ring, C–O, and C–H stretching vibrations, which are the key structural features for confirming the esterification reaction. The FTIR spectrum in Figure 1 confirms the successful synthesis of AI-2959 through characteristic absorption peaks. The broad peak at 3472.50 cm^−1^ corresponds to –OH stretching vibrations. Peaks at 3079.72 cm^−1^ and in the range of 612.31–849.54 cm^−1^ confirm the presence of aromatic rings, while absorptions at 2934.46 cm^−1^ and 2870 cm^−1^ are attributed to C-H stretching vibrations from -CH_2_- and -CH_3_ groups. The strong carbonyl stretching vibration at 1726.86 cm^−1^ represents the newly formed ester bond between aspirin and I-2959. Additionally, the C-O stretching vibrations at 1256.93 cm^−1^ and 1163.65 cm^−1^ confirm the presence of an ester group and tertiary alcohol, respectively. These spectral features collectively demonstrate the successful esterification reaction.

### 3.2. Proton Nuclear Magnetic Resonance (^1^H NMR) Analysis

**Figure 2:**
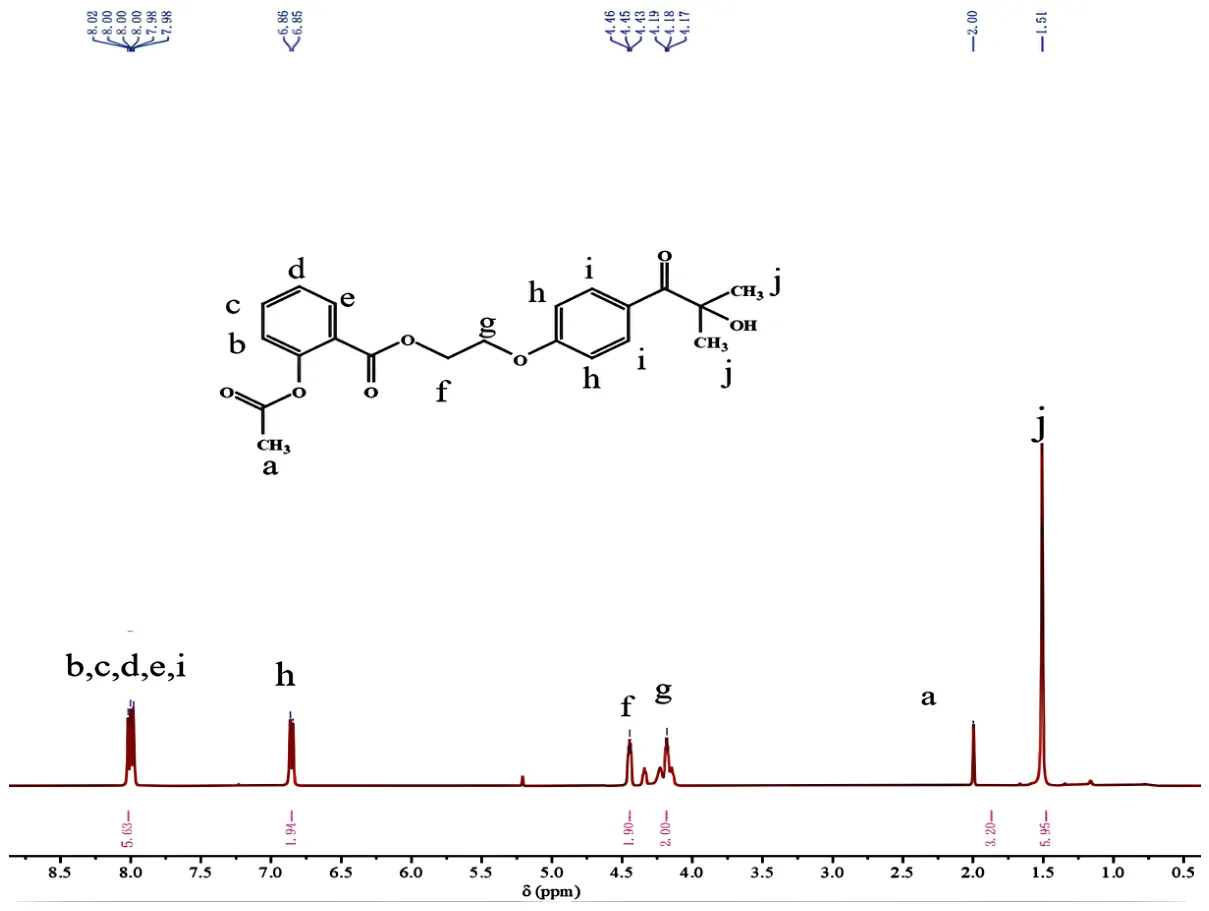
^1^HNMR spectrumofAI-2959.

The structure of the synthesized AI-2959 was confirmed through ^1^H NMR spectroscopy. The analysis was performed at room temperature using an AVANCE III ^1^H NMR spectrometer (Bruker 400MHz, Germany), with CDCl_3_ as the solvent. The spectrum in Figure 2 displays distinct proton signals that confirm the molecular structure. The multiplet at δ = 8.09–7.94 ppm corresponds to the aromatic protons on the ortho-substituted benzene ring. The signal at δ = 6.85 ppm is assigned to the aromatic protons adjacent to the ether linkage. The methylene groups observed at δ = 4.45 ppm and δ = 4.18 ppm are connected and adjacent to the ester group, respectively. A singlet at δ = 2.00 ppm is attributed to the methyl group of the acetyl moiety, and the singlet at δ = 1.51 ppm corresponds to the methyl groups adjacent to the hydroxyl group. These signals collectively verify the successful formation of the AI-2959 structure through esterification.

### 3.3. Elemental Analysis

An Elementar UNICUBE elemental analyzer (Elementar Co., Ltd., Langenselbold, Germany) was used. The sample burns and decomposes at high temperatures, converting the elements under study into gaseous products; separation was performed using a TPD-type temperature-programmed desorption, followed by detection in a TCD thermal conductivity detector, which was performed to determine the content of C, H, and O in AI-2959. In Table 1, the measured contents of each element are provided, and the values are in good agreement with the theoretical values calculated for the molecular formula C_21_H_22_O_7_.

### 3.4. UV-Vis Spectroscopy Analysis

**Figure 3:**
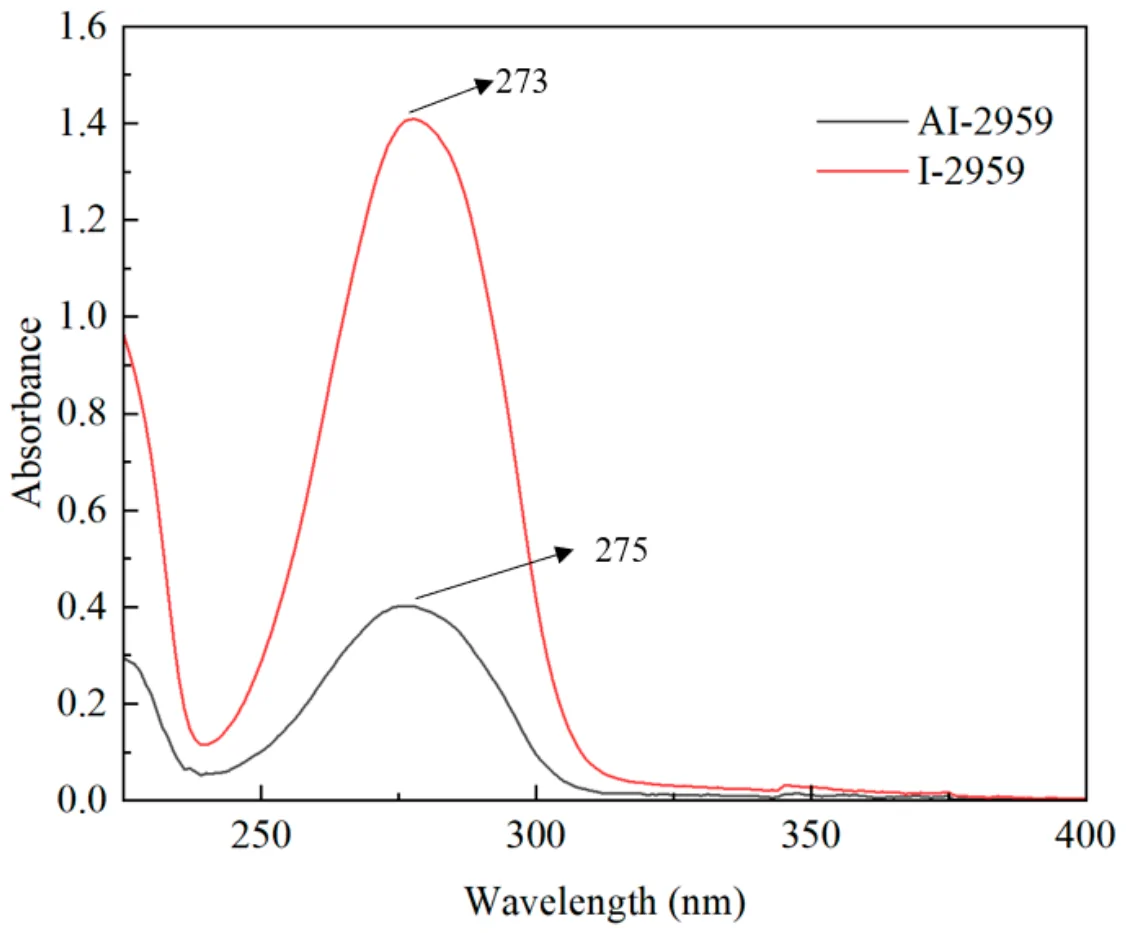
UV-Vis spectra of I-2959 and AI-2959 at concentrations of 5 × 10^−5^ mol/Land 1.5 × 10^−5^ mol/L, respectively.

The UV-Vis absorption spectra of I-2959 and AI-2959, determined in acetonitrile, are shown in Figure 3. AI-2959 exhibits a maximum absorption wavelength (λ_max_) at 275 nm, which is very similar to the λ_max_ of I-2959 at 273 nm. The strong absorption bands observed below 250 nm are mainly attributed to high-energy electronic transitions, including π→π* transitions associated with the aromatic rings and ester groups in both photoinitiators. These absorption features reflect the intrinsic electronic structures of I-2959 and AI-2959. Although these short-wavelength absorptions contribute to the overall UV absorption behavior, they are not the dominant absorption region for the 365 nm UV curing process. To eliminate the influence of concentration difference, the molar extinction coefficient (ε_max_) was calculated according to the Lambert–Beer law (A = *εbc*) for quantitative comparison of intrinsic light absorbing capacity. As shown in Table 2, AI-2959 shows a maximum absorption wavelength λ_max_ of 275 nm, close to 273 nm for I-2959. The molar extinction coefficient ε_max_ of AI-2959 reaches2.68 × 10^4^L/mol·cm, higher than 2.53 × 10^4^ L/mol·cm of I-2959. This result demonstrates that AI-2959 possesses a stronger intrinsic light absorbing ability at an identical molar concentration, which implies improved photosensitivity in UV curing systems.

### 3.5. Thermogravimetric Analysis (TGA)

**Figure 4:**
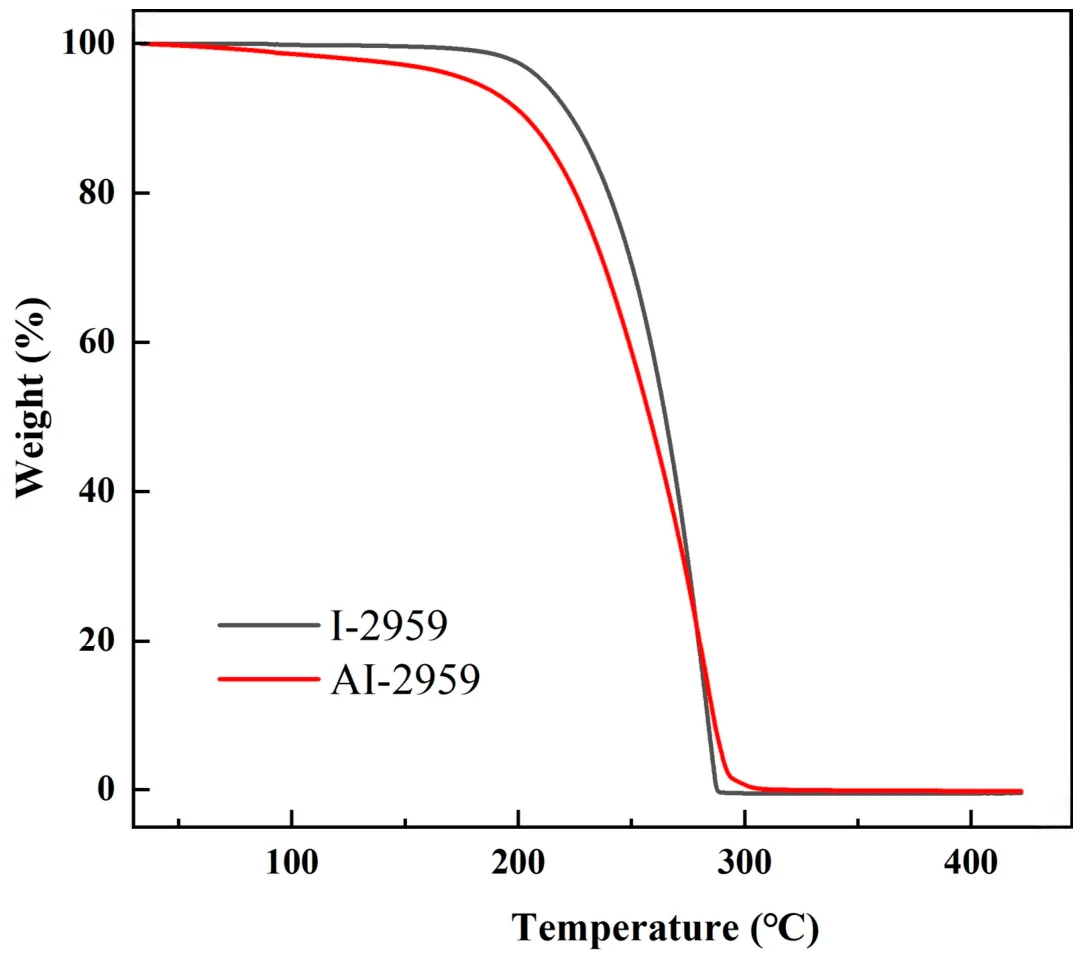
TG spectra of I-2959 and AI-2959.

The thermal stability of I-2959 and AI-2959 was assessed by TGA under a nitrogen atmosphere (Figure 4). The results show that AI-2959 undergoes 5% weight loss at 178 °C and complete degradation at 292 °C. In comparison, I-2959 exhibited 5% weight loss at a higher temperature (210 °C) but degraded completely at a slightly lower temperature (287 °C). The degradation of AI-2959 occurred in two stages: an initial low-temperature stage involving the release of water molecules and the aspirin moiety, followed by the main degradation process between 201 °C and 292 °C. The TG profile of AI-2959 demonstrated a more gradual degradation and slightly higher final decomposition temperature, indicating improved thermal stability over I-2959.

### 3.6. Photopolymerization Kinetics

**Figure 5:**
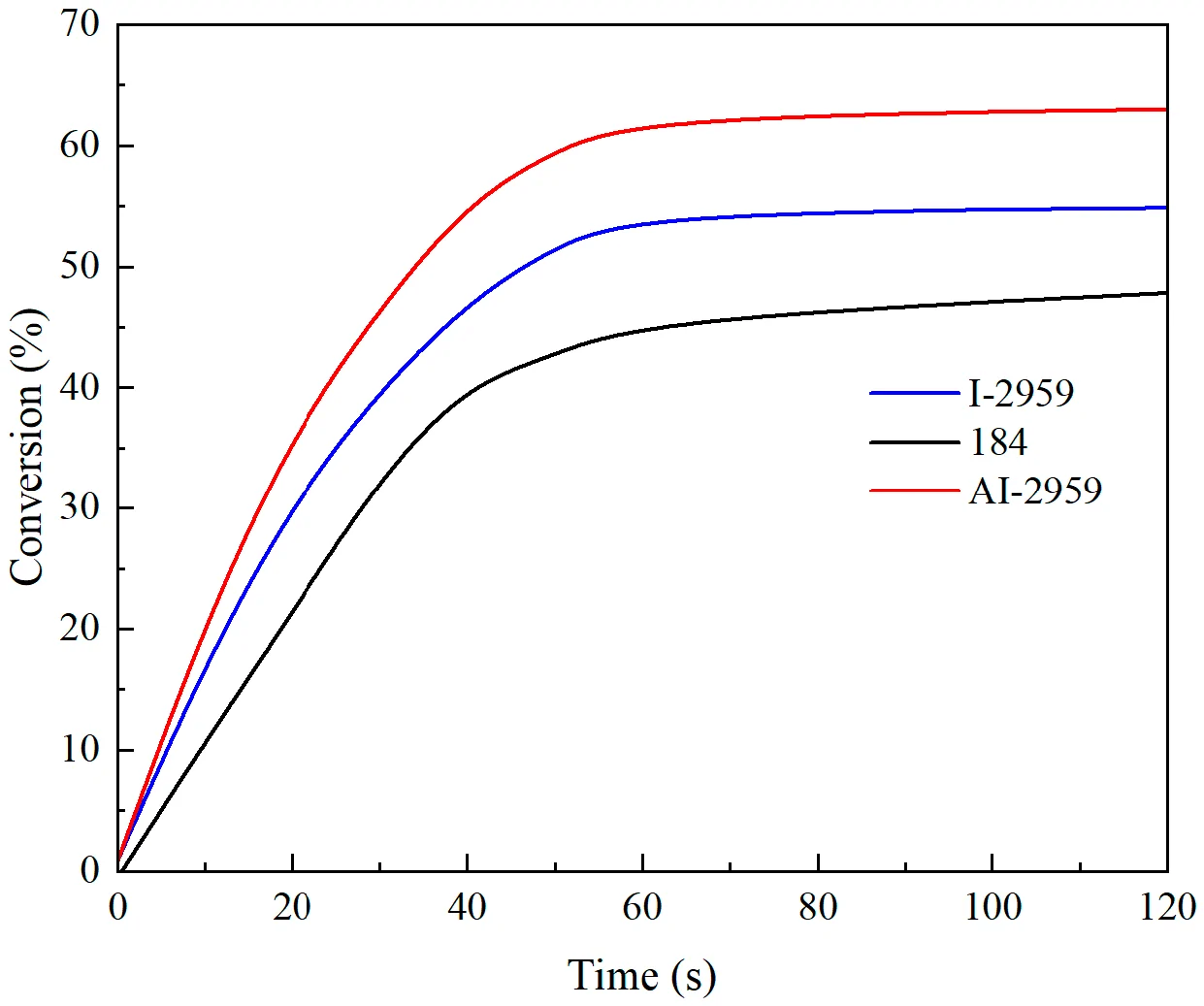
Conversion rate curves of 184, I-2959, and AI-2959 upon induction of TMPTA photopolymerization.

The photopolymerization kinetics of TMPTA initiated by AI-2959, I-2959, and photoinitiator 184 were investigated by RT-FTIR analysis. The double-bond conversion was calculated from the decrease in the acrylate C=C absorption peak according to Equation (3) described in Section 2.6. In Figure 5, the photopolymerization kinetics analysis of TMPTA revealed a typical three-stage conversion profile, consisting of a rapid initial reaction period (10–30 s) where the polymerization rate peaked, followed by a deceleration period (40–60 s) and a plateau (60–120 s) where the conversion rate change was less than 0.5%/s. After 120 s of irradiation, the final double-bond conversion rates achieved were 44.2% for the system initiated with conventional photoinitiator184, 56.1% for I-2959, and 61.3% for AI-2959. The superior performances of I-2959 and AI-2959 were attributed to the α-hydroxy ketone group, which has a high quantum yield for Norrish Type I photoscission. Furthermore, the extended molecular chain of AI-2959 likely reduces the oxygen diffusion coefficient, enhancing its resistance to oxygen inhibition, as compared with the I-2959 system.

The enhanced photopolymerization performance of AI-2959 can be explained from both molecular structure and photophysical aspects. The α-hydroxy ketone moiety retained in AI-2959 is responsible for efficient Norrish Type I photocleavage under UV irradiation, generating active radical species to initiate polymerization. Compared with I-2959, the introduction of the acetylsalicylic acid segment increases the molecular conjugation and polarity of AI-2959, resulting in a higher molar extinction coefficient and improved photon absorption efficiency.

In addition, the enlarged molecular structure of AI-2959 increases steric hindrance and reduces the diffusion of oxygen molecules into the curing system. Since oxygen inhibition is mainly caused by radical scavenging reactions between oxygen and active radicals, the reduced oxygen permeability contributes to faster polymerization initiation and higher final conversion.

Therefore, the improved curing performance of AI-2959 is attributed to the synergistic effects of enhanced light absorption, efficient radical generation from the α-hydroxy ketone group, and improved resistance toward oxygen inhibition.

**Figure 6:**
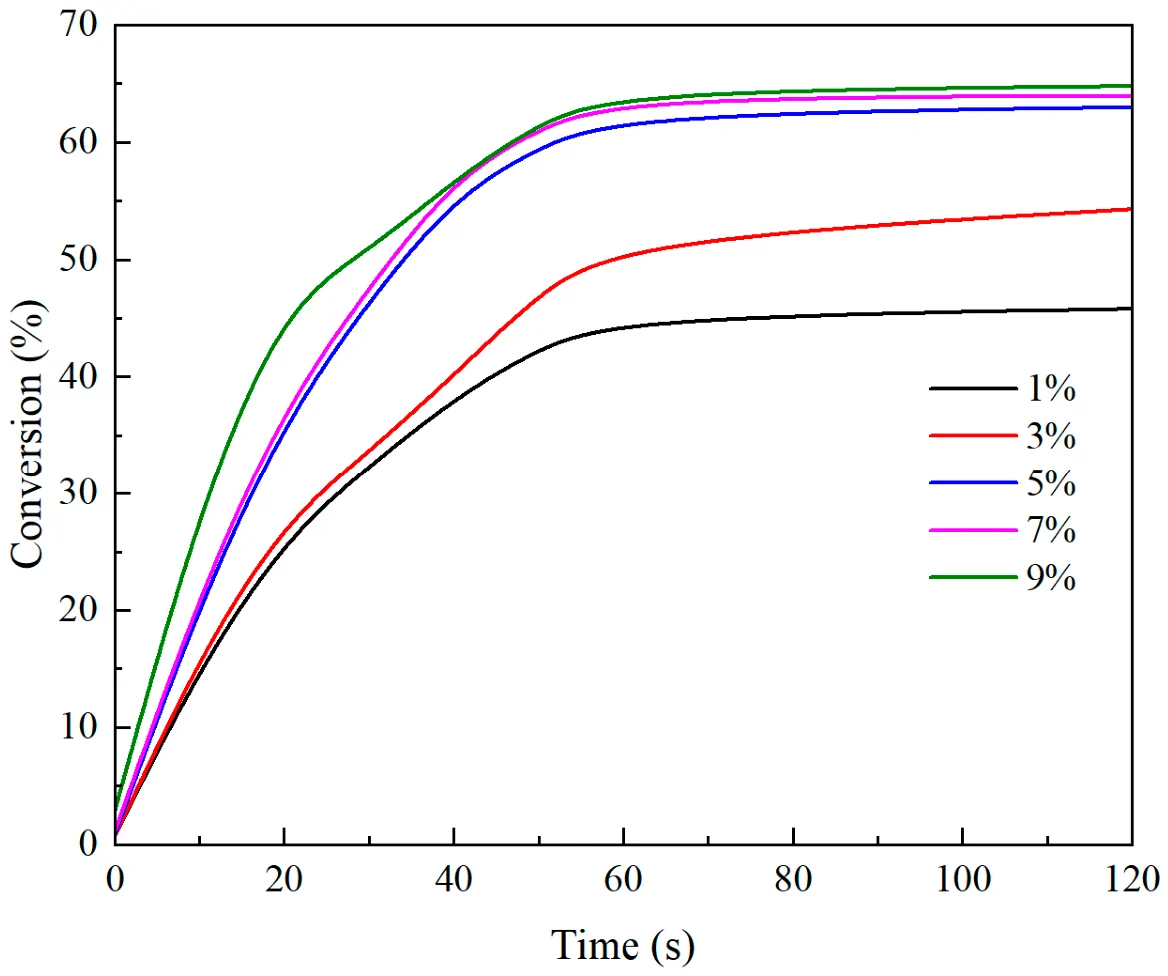
Photopolymerization curves with 1%, 3%, 5%, 7%, and 9% wt% concentrations of AI-2959.

The polymerization efficiency was also evaluated for different concentrations of AI-2959, ranging from 1% to 9% (wt.%). Figure 6 shows that 5% AI-2959 achieved a final conversion of 61%. Increasing the concentration to 9% only marginally improved the conversion by 0.3% but increased the initiator residue by 23%. Thus, 5% was the optimal AI-2959 concentration, in terms of cost and performance. The concentration-dependent behavior indicates that increasing AI-2959 content initially provides more active radical species and accelerates polymerization. However, excessive photoinitiator concentration does not significantly improve conversion because radical recombination and light shielding effects may occur at higher concentrations. Therefore, 5 wt.% AI-2959 provides an appropriate balance between initiation efficiency and material performance.

### 3.7. Coating Properties

The curing performance and coating properties are summarized in Table 3 and Table 4. The effect of AI-2959 concentration on curing efficiency (Table 3) reveals an optimal loading of 5 wt.%. When the concentration increased from 1% to 5%, the curing time sharply decreased from 26 s to 14 s, attributed to the increased availability of reactive radicals for initiating polymerization. However, further increasing the concentration to 7% or 9% did not yield additional acceleration. This plateau is ascribed to the internal filter effect (light screening) and enhanced radical recombination at high photoinitiator concentrations, where excess radicals terminate prematurely rather than initiating propagation, thereby providing no net benefit to curing speed. Therefore, 5 wt.% AI-2959 was identified as the optimal concentration for subsequent comparative studies.

A comparison of the three photoinitiator systems (Table 4) provides further mechanistic insights into the performance advantages of AI-2959. Under a medium-pressure mercury lamp (50 mW/cm^2^), the AI-2959 system (5% loading) cured in 14 s, which was 17.6% faster than photoinitiator 184 (17 s) and 6.7% faster than I-2959 (15 s). The superior curing speed of AI-2959 correlates well with its higher molar extinction coefficient (2.68 × 10^4^ L mol^−1^ cm^−1^) and enhanced resistance to oxygen inhibition, as discussed in Section 3.6. The enhanced photon absorption efficiency of AI-2959 enables the faster generation of initiating radicals, while its larger molecular structure reduces oxygen diffusion into the curing system, collectively accelerating the polymerization process.

The notably higher pencil hardness of the AI-2959 system (5H), compared with 4H for I-2959 and 3H for photoinitiator 184, can be rationalized by two factors: (i) the higher double-bond conversion (61.3%) achieved with AI-2959 leads to a more densely crosslinked polymer network, and (ii) the rigid acetylsalicylic acid moiety incorporated into the photoinitiator structure imparts additional stiffness to the cured matrix, enhancing the overall mechanical resistance of the coating.

The adhesion performance, classified as Grade 1B for AI-2959 versus 0B for both I-2959 and photoinitiator 184, indicates significantly improved interfacial bonding. This enhancement is attributed to the increased polarity of AI-2959 resulting from the ester and aromatic groups introduced by the aspirin moiety, which promotes better wetting, spreading, and adhesion to the polar glass substrate. The improved adhesion further supports the potential of AI-2959 for practical coating applications where strong interfacial bonding is required.

All three systems exhibited good flexibility and acid resistance (1 M HCl, 24 h), resulting in smooth, uniform, and transparent films without visible defects, confirming good compatibility between the photoinitiators and the TMPTA matrix. The cured films prepared using AI-2959, I-2959, and photoinitiator 184 exhibited similar visual appearances, indicating that the introduction of the aspirin moiety does not adversely affect film formation or optical clarity. However, none of the systems demonstrated good alkali resistance (1 M NaOH, 24 h), which is attributed to the susceptibility of ester bonds in both the photoinitiator structures and the TMPTA polymer network to alkaline hydrolysis. This limitation suggests that the current formulations are more suitable for applications where alkaline exposure is not anticipated, and it highlights a direction for future optimization, such as introducing hydrolysis-resistant crosslinkers or exploring alternative monomer systems.

### 3.8. Small-Molecule Migration Analysis

**Figure 7:**
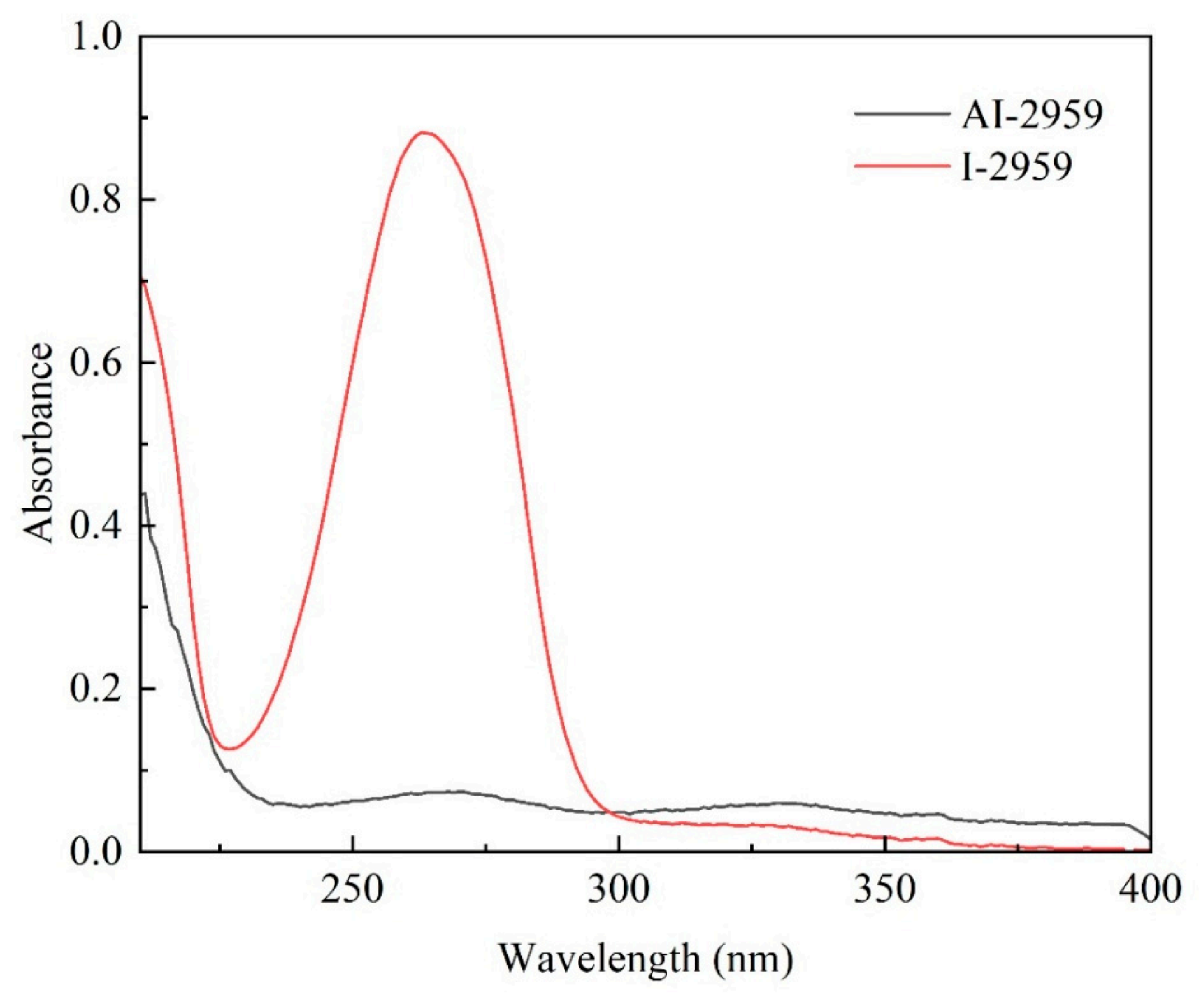
UV-Vis absorption spectra of extraction solution from AI-2959 and I-2959.

The migration of the unreacted photoinitiator was assessed by extracting cured films with acetonitrile and analyzing the extract via UV-Vis spectroscopy. In Figure 7, the spectra show characteristic absorption peaks at λ_max_ = 273 nm for I-2959 and λ_max_ = 275 nm for AI-2959, confirming the presence of residual, migratable PI in both cured systems. Beyond 300 nm, both extraction spectra show negligible absorption, indicating that no additional absorbing species were generated during UV curing or extraction. The absence of significant absorption in this region further confirms that the detected signals mainly originate from residual I-2959 and AI-2959 molecules rather than degradation products or other impurities. Table 5 provides the calculated relative migration rate (R) of AI-2959, which was only 10.2% of that measured for I-2959. This dramatic reduction was primarily attributed to the 43.8% increase in the molecular weight of AI-2959 (MW = 386.4 g/mol) compared with I-2959 (MW = 256.3 g/mol). This results in a lower diffusion coefficient and makes AI-2959 highly suitable for applications such as biocompatible materials, where low migration is critical.

## 4. Conclusions

In conclusion, a novel modified photoinitiator AI-2959 was successfully synthesized through esterification of I-2959 with acetylsalicylic acid. Structural characterization confirmed the successful introduction of the aspirin-derived moiety while maintaining the α-hydroxy ketone photoreactive group. Compared with I-2959, AI-2959 exhibited a higher molar extinction coefficient, improved thermal stability, enhanced resistance to oxygen inhibition, and higher photopolymerization efficiency.

In TMPTA photopolymerization, AI-2959 achieved a double-bond conversion of 61.3% after 120 s irradiation, exceeding I-2959 and PI 184. Furthermore, AI-2959 showed faster curing, higher hardness, improved adhesion, and significantly reduced migration behavior. These results demonstrate that molecular modification provides an effective strategy to simultaneously improve photoinitiation efficiency and safety characteristics, indicating the potential application of AI-2959 in UV-curable biomedical materials and pharmaceutical formulation.

## Figures and Tables

**Table 1:** Elemental composition of AI-2959.

Element	C	H	O
Theoretical	65.28%	5.74%	28.98%
Measured	63.88%	6.22%	29.90%

**Table 2:** Comparison of the maximum absorption wavelength and molar extinction coefficient of I-2959 and AI-2959.

Photoinitiator	λ_max_/nm	A	ε_max_/(L/mol·cm)
I-2959	273	1.408	2.53 × 10^4^
AI-2959	275	0.4022	2.68 × 10^4^

**Table 3:** Curing times of coatings with different AI-2959 concentrations.

AI-2959 Content (wt.%)	1	3	5	7	9
Curing time (s)	26	18	14	14	14

**Table 4:** The film properties from different photoinitiators of 184, I-2959, and AI-2959.

	AI-2959	Photoinitiator 184	I-2959
Curing time (s)	14	17	15
Hardness (H)	5	3	4
Adhesion (B)	1	0	0
Flexibility	Good	Good	Good
Acid resistance ^a^	Not whitish or foggy	Not whitish or foggy	Not whitish or foggy
Alkali resistance ^b^	Poor	Poor	Poor
Coating appearance	Smooth, transparent	Smooth, transparent	Smooth, transparent

^a^, (1 M HCl, soaked for 24 h); ^b^, (1 M NaOH, soaked for 24 h).

**Table 5:** Relative migration of AI-2959 and I-2959.

Samples	A (λmax)	c (mol/L)	R (%)
I-2959	0.8814	3.4 × 10^−5^	100
AI-2959	0.0734	2.68 × 10^−5^	10.2

## Data Availability

The original contributions presented in this study are included in the article. Further inquiries can be directed to the corresponding author.
